# Implant contamination as a cause of surgical site infection in spinal surgery: are single-use implants a reasonable solution? – a systematic review

**DOI:** 10.1186/s12891-020-03653-z

**Published:** 2020-09-25

**Authors:** Friederike Schömig, Carsten Perka, Matthias Pumberger, Rudolf Ascherl

**Affiliations:** Center for Musculoskeletal Surgery, Charité – University Medicine Berlin, Charitéplatz 1, 10117 Berlin, Germany

**Keywords:** Implant contamination, Sterilization, Single-use implants, Spinal surgery, Bacteria

## Abstract

**Background:**

In spine surgery, surgical site infection (SSI) is one of the main perioperative complications and is associated with a higher patient morbidity and longer patient hospitalization. Most factors associated with SSI are connected with asepsis during the surgical procedure and thus with contamination of implants and instruments used which can be caused by pre- and intraoperative factors. In this systematic review we evaluate the current literature on these causes and discuss possible solutions to avoid implant and instrument contamination.

**Methods:**

A systematic literature search of PubMed addressing implant, instrument and tray contamination in orthopaedic and spinal surgery from 2001 to 2019 was conducted following the PRISMA guidelines. All studies regarding implant and instrument contamination in orthopaedic surgery published in English language were included.

**Results:**

Thirty-five studies were eligible for inclusion and were divided into pre- and intraoperative causes for implant and instrument contamination. Multiple studies showed that reprocessing of medical devices for surgery may be insufficient and lead to surgical site contamination. Regarding intraoperative causes, contamination of gloves and gowns as well as contamination via air are the most striking factors contributing to microbial contamination.

**Conclusions:**

Our systematic literature review shows that multiple factors can lead to instrument or implant contamination. Intraoperative causes of contamination can be avoided by implementing behavior such as changing gloves right before handling an implant and reducing the instruments’ intraoperative exposure to air. In avoidance of preoperative contamination, there still is a lack of convincing evidence for the use of single-use implants in orthopaedic surgery.

## Background

In the past few years, the number of spine surgeries performed worldwide has been steadily increasing [1, 2]. One of the main perioperative complications in orthopaedic surgery is surgical site infection (SSI). In spinal surgery, surgical site infection occurs in 2 to 13% and is associated with an increase in patient morbidity, revision surgery, extended hospitalization and health care costs [3, 4]. Factors associated with SSI are the procedures performed, surgical environment and technique, reprocessing procedures of implants and instruments, postoperative measures, and the patients’ immunity. Most of these factors rely on asepsis rather than increasing or decreasing a patient’s immunity [5].

For surgical procedures, the reprocessing of medical products such as surgical instruments or implants by the sterilization processing department is a key process in standard clinical practice and is thought to be essential in the prevention of SSI. As part of this process, surgical instruments and implants are decontaminated, washed, reassembled, labelled, sterilized and redistributed. Since only a small portion of processed implants is used during surgery, these implants are reprocessed multiple times before surgically being implanted in a patient. Even though this is standard clinical practice, little is known about the long-term behavior of reprocessed products especially regarding their contamination. Additionally, despite efforts to reduce the risk of contamination during surgery, correct handling of implants and instruments continues to constitute a challenge and thereby a possible threat to patient safety.

Multiple studies have been conducted on the causes of implant and instrument contamination. Based on their findings, these causes can be divided into two groups: intraoperative contamination for example via air or gloves versus preoperative contamination due to inadequate reprocessing. We conducted this systematic review to evaluate these sources of implant contamination and discuss possible solutions both pre- and intraoperatively to avoid implant and instrument contamination, which in turn leads to higher patient safety during surgery.

## Methods

In September 2019 we conducted a systematic literature search of PubMed addressing implant, instrument and tray contamination in orthopaedic and spinal surgery. The systematic review has been reported in accordance with the PRISMA statement [6]. See Table 1 for search terms used. Inclusion criteria comprised studies published in English and studies performed on humans in vivo. Studies in other fields than orthopaedics were excluded as well as studies which did not focus on causes of contamination. Case reports, review articles, technical notes, opinions of experts, and letters to the editors were also excluded. The selected studies’ abstracts were screened and if found inadequate, the full text was evaluated.

**Table 1:** Search strategy

Search #	Query
#1	Implant or instrument or tray and contamination and orthopedics
#2	Implant or instrument or tray and contamination and spine
#3	Implant or instrument or tray and contaminated and orthopedics
#4	Implant or instrument or tray and contaminated and spine
#5	#1 or #2 or #3 or #4

## Results

**Fig. 1:**
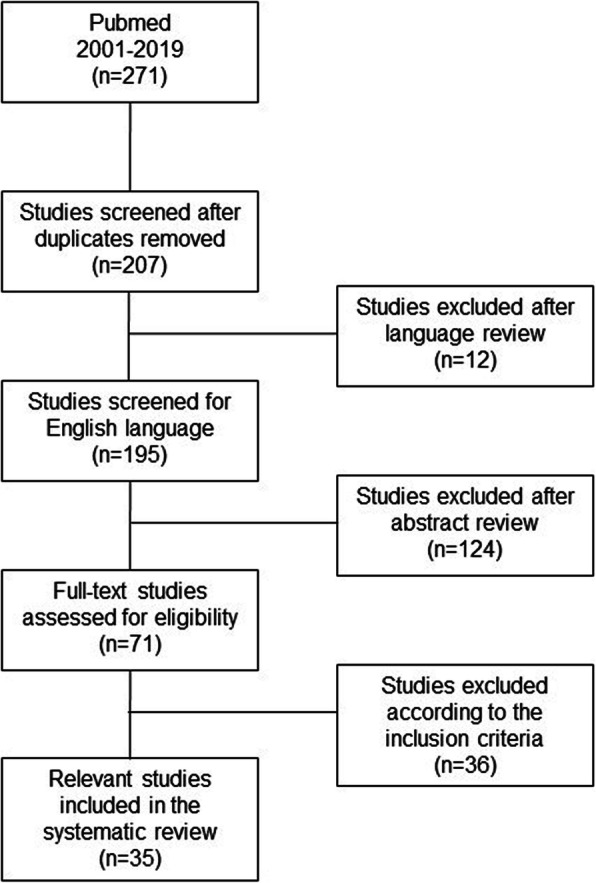
Flow chart of the literature research using the PRISMA guidelines

A flow chart of our literature research was created using the PRISMA guidelines (Fig. 1). We identified 271 potential studies via our search strategy. Fifty-four studies were duplicates and thus excluded. Another 12 studies were excluded because they were not written in English. We then excluded 124 studies after reviewing title and abstract. The 71 remaining studies were then assessed for eligibility. Finally, we selected 35 studies for inclusion in our systematic review and divided them into pre- and intraoperative causes of implant and instrument contamination (Tables 2 and 3).

**Table 2:** Included studies on preoperative sources of implant contamination

Study	Source of contamination	Study size	Main results	Conclusion
**Preoperative sources of implant contamination**				
Agarwal et al. (2019a) [7]	Pedicle screws	1. 6 pedicle screws 2. 1 implant tray with 164 pedicel screws	1. 3 types of contaminants: corrosion, saccharide of unknown origin, soap residue 2. observed reprocessing time was < 2 h	Repeatedly reprocessed pedicle screws may be source of SSI
Pinto et al. (2010) [8]	Surgical implants	227 samples (76 from clean surgeries, 76 from contaminated surgeries, 75 from infected surgeries)	47% microbial contamination of implants in clean surgeries, 70% in contaminated, and 80% in infected surgeries	Most of the microorganisms recovered are covered by the cleaning and sterilization process; antibiotic prophylaxis is important in clean surgeries
Lopes et al. (2019) [9]	FMRs and DGs	9 FMRs and 9 DGs (3 rinsing, 3 manual cleaning, 3 manual plus automated cleaning)	100% ATP contamination in rinsed only with 2–2.5 log_10_ fold reduction after manual or manual plus automated cleaning; soil present in all groups	Reusable surgical instruments show residual biological soil after reprocessing, which may have an adverse effect on patient outcome
Costa et al. (2018) [10]	FMRs, DGs, and single-use screws in clinical use for > 1 year	73 FMRs (16 ATP, 8 CFU, 40 visual, 9 SEM), 19 DGs (8 ATP, 8 visual, 3 SEM), 123 screws (24 CFU, 90 visual, 9 SEM)	1. FMRs: 75% showed ATP, 85% visible soil, 63% protein after cleaning 2. DGs: 38% showed ATP, 100% soil after cleaning 3. Screws: Biofilm and soil were visible after cleaning	Ineffectiveness of manual reprocessing and reprocessing practices threatens patient safety
Smith et al. (2018) [11]	Surgical drills	15 cannulated drill bits (3 per group)	2 negative controls showed contaminant bacteria; 1 experimental drill showed inoculation bacteria	Standard autoclave sterilization may be inefficient and delay of reprocessing may increase the risk of resistant contamination
Mayer et al. (2016) [12]	Femoral BHs	2 femoral BHs	Complete eradication at all target locations	Adequate decontamination of BHs can be achieved after steam sterilization
Bundgaard et al. (2019) [13]	Scissors, knife shafts, puncture cannulae	Not clear	All sterilized instruments showed protein residues below the accepted threshold regardless of holding time	No association between residual protein and holding time
Mont et al. (2013) [14]	Saws, cutting guides, trays	202 patients treated with conventional instruments, 205 patients treated with single-use instruments	Single-use instrumentation led to a significant reduction of compromised pans from 7 to 1%; decrease in contamination in 57%	Single-use instruments will play an increasing role in orthopaedic surgery

*BH* broach handle, *CFU* colony forming unit, *DG* depth gauge, *FMR* flexible medullary reamer, *SEM* scanning electron microscopy

**Table 3:** Included studies on intraoperative sources of implant contamination

Study	Source of contamination	Study size	Main results	Conclusion
**Intraoperative sources of implant contamination**				
**Surgical instrument trays**				
Waked et al. (2007) [15]	Surgical instrument trays	90 sterilization wraps	Detection rates ranged from 7 to 97%	Substantial perforations in sterilization wraps may be missed
Mobley and Jackson 3rd. (2018) [16]	Surgical instrument trays	20 sterilization wraps	Overall 56% accuracy	Current method for assessing sterility is inadequate
**Surgical equipment**				
Radcliff et al. (2013) [17]	Preoperative in-room time	7991 spine surgeries including 276 SSIs	ART was significantly higher in patients with infection (68 vs. 61 min); significant increase in infection rate if ART was > 1 h	Preoperative in-room time is a risk factor for SSI
Blom et al. (2000) [18]	Surgical drapes	24 agar plates covered with 7 types of surgical drapes	All of the reusable woven drapes allowed bacterial penetration; non-woven drapes were impermeable apart from one	Recommendation for non-woven disposable drapes over woven drapes
Lankester et al. (2002) [19]	Surgical gowns	40 surgical gowns of 2 types	Disposable gowns showed less bacterial penetration than reusable gowns in all tested regions	Reusable gowns may be unsuitable for use in orthopaedic implant surgery
Ward Sr et al. (2014) [20]	Surgical gloves and gowns	1. 102 surgical team members 2. 251 surgical team members	1. 31 vs. 7% baseline bacterial contamination in cloth gowns vs. paper gowns 2. 23% of surgeons retaining outer gloves had positive glove contamination vs. 13% of those exchanging gloves	Recommendation for disposable paper gowns and outer glove exchange just before handling implant materials
Klaber et al. (2019) [21]	Surgical gowns	140 surgical gowns	Bacterial contamination in 12% of surgical gowns (4% in total hip arthroplasty vs. 22% in spine and knee surgery)	Higher surgical gown contamination during non-arthroplasty procedures
Wichmann et al. (2019) [22]	Surgical gloves	43 pairs of knitted cotton outer gloves	9% of gloves yielded > 100 CFU under aerobic conditions, 14% under anaerobic conditions	Low microbial contamination of knitted cotton outer gloves, but relevant proportion showing contamination above minimal thresholds
Amirfeyz et al. (2007) [23]	Theatre shoes	50 outside shoes, 50 theatre shoes morning and 50 end of day	Microbial growth in 90% of outside shoes, 68% of theatre shows in the morning, and 56% of theatre shoes end-of-day	Recommendation for dedicated theatre shoe use and good floor washing protocol
**Implant exposure to air**				
Bible et al. (2013) [24]	Coverage of implants	105 surgical trays (54 uncovered vs. 51 covered trays)	Overall 10% contamination with 2% of covered vs. 17% of uncovered implants	Coverage of implants significantly reduces their contamination
Dalstrom et al. (2008) [25]	Coverage of implants	45 surgical trays (15 uncovered and no traffic, 15 uncovered and traffic, 15 covered)	Microbial growth in 4% at 30 min to 30% at 4 h of uncovered trays vs. 0% in covered trays	Coverage of implants significantly reduces their contamination; microbial growth correlated with the duration of open exposure
Menekse et al. (2015) [26]	Coverage of implants	42 surgical trays (20 uncovered vs. 22 covered)	Microbial growth in 55% vs. 18% in uncovered and covered trays, respectively, after 120 min	Coverage of implants significantly reduces their contamination; microbial growth correlated with the duration of open exposure
Uzun et al. (2019) [27]	Coverage of implants	60 surgical trays (30 uncovered vs. 30 covered)	Statistically significant difference in contamination at all time points	Coverage of implants significantly reduces their contamination; microbial growth correlated with the duration of open exposure
Agarwal et al. (2019b) [28]	Usage of an impermeable guard	10 sterile packaged pedicle screws (5 with and 5 without an intraoperative guard)	All samples without guard showed bacterial growth; none with guard	Using an intraoperative guard provides higher asepsis
Smith et al. (2009) [29]	Individual packaging	50 screw packets	Microbial growth on 48% of packet exteriors and in 14% of acts of opening	Individual packaging of screws is a potential risk factor for contamination
**Surgical environment**				
Andersson et al. (2012) [30]	Door openings, number of persons in the OR	30 orthopaedic surgeries in 3 ORs	Positive correlation between CFU and door openings and CFU and number of persons in the OR	Negative impact of traffic flow and number of persons present in the OR
Perez et al. (2018) [31]	Door openings, number of persons in the OR	48 orthopaedic and general surgeries	Positive correlation between CFU and door openings and number of persons in the OR	Negative impact of traffic flow and number of persons present in the OR
Knobben et al. (2006) [32]	Door openings, number of persons in the OR, airflow systems	207 orthopaedic surgeries	Under original conditions 33% of contamination and 11% of SSI, after disciplinary measures and LAF installation 9 and 1% of SSI	Systemic and behavioral changes significantly decrease bacterial contamination and SSI
Andersson et al. (2014) [33]	Airflow systems	63 orthopaedic implant surgeries (30 DV, 33 LAF)	Bacterial growth > 10 CFU/m^3^ in 1% of LAF ORs and 57% of DV ORs	LAF ORs offer high-quality air during surgery
Sadrizadeh et al. (2014) [34]	Airflow systems	Physical model	Reduction of airborne and sedimenting bacteria-carrying particles by MLAF	MLAF may be an option to reduce the level of microbial contamination
Sossai et al. (2011) [35]	Airflow systems	34 total knee arthroplasties (17 with MLAF, 17 without)	Reduction of bacterial count from 24 CFU/m^3^ without MLAF to 4 CFU/m^3^ with MLAF	MLAF may be an option to reduce the level of microbial contamination
Noguchi et al. (2017) [36]	Airborne particles	3 patterns of physical movements	Large number of particles when unfolding surgical gown, removing gloves, and putting arms through gown sleeves; LAF reduced particles	Unnecessary actions should be avoided and LAF potentially reduces bacterial contamination
Richard and Bowen (2017) [37]	OR surfaces	13 surfaces in 6 orthopaedic ORs	Bioburden detectable on all included surfaces	Detection of environmental trouble spots in the OR possible with ATP bioluminescence
**Supportive equipment**				
Ahmad et al. (2011) [38]	Supports	40 supports used in 20 hip arthroplasty procedures	85% of anterior and 50% of posterior supports showed bacterial colonisation	High bacterial load on supports may contribute to higher infection rates
Ranawat et al. (2004) [39]	Pressure sore prevention pads	13 pressure sore prevention pads	85% of pads showed bacterial growth	Use of pressure sore prevention pads should be closely reviewed
Ahmed et al. (2009) [40]	Tourniquets	20 tourniquets	All tourniquets were contaminate with 9 to > 385 CFU	Tourniquets should be cleaned before every surgery

*ART* anesthesia ready time, *CFU* colony forming units, *DV* displacement ventilation system, *LAF* laminar airflow ventilation system, *MLAF* mobile laminar airflow ventilation system, *OR* operating room, *SSI* surgical site infection

### Preoperative sources of implant contamination

Agarwal et al. recently published a study examining the presence of residual non-microbial contaminants and/or foreign material in pedicle screws after they had been sterilized. Optical microscopy, scanning electron microscopy with energy dispersive spectroscopy, and Fourier transform infrared spectroscopy were used in order to identify contaminants on six pedicle screws from four different trays of sterilized implants. In this study, corrosion, saccharides, soap residues and salt residues were found on the examined pedicle screws. According to the authors, finding saccharides was associated with biofilm and the presence of endotoxins. In a second step, the authors analyzed the manufacturer’s guideline regarding the reprocessing recommendations and compared these to their real-time observation of the procedure. Interestingly, the manufacturer recommended at least 19 man-hours for reprocessing of an implant tray with 164 pedicle screws whereas the actually observed reprocessing time was less than 2 hours [7].

Other recent studies also focus on this problem of non-microbial as well as microbial contamination of surgical implants. The importance of reprocessing was emphasized by Pinto et al., who showed in 227 samples that 47% of surgical instruments were contaminated after clean surgery, 70% after contaminated surgery, and 80% after infected surgery [8]. In a smaller study, Lopes et al. investigated the problem of implant contamination after reprocessing by contaminating different reusable surgical instruments with sheep blood and *Staphylococcus aureus* before repeating different cleaning methods 20 times. Afterwards, they measured adenoside triphosphate (ATP), the microbial load, residual protein as well as soil and biofilm presence and were able to show that independent of the cleaning methods used soil or biofilms were evident on the cleaned surgical instruments [9]. Similar results were shown by Costa et al. who assessed 215 surgical instruments, which had been in clinical use for over a year, for residual ATP, protein, bacterial contamination, endotoxin and biofilm. After sterilization, biofilm and soil were still detectable by electron microscopy [10]. A study by Smith et al. implicated that standard sterilization of cannulated drills may ineffectively sterilize cannulated drills and thus lead to bacterial contamination of these instruments [11]. However, Mayer et al. showed in two femoral broach handles that an adequate decontamination of these instruments can be achieved in both the disassembled and the assembled state [12]. Bundgaard et al. also did not identify protein residues over the threshold even after prolonged holding times of used medical equipment [13]. In knee arthroplasty, it was shown that a decrease in contamination was achieved when using single-use instead of reusable instruments, which in most cases was due to fewer torn wraps in the single-use group [14].

### Intraoperative sources of implant contamination

Incorrect or insufficient reprocessing of implants and instruments is not the only source of medical products’ contamination and thereby surgical site infection. The common practice of operating room (OR) personnel evaluating sterile wraps for breaches before using contained instruments is questioned by Waked et al., who found that even bigger wrap defects are missed in 18% [15]. More than 10 years later, Mobley and Jackson showed similar results with an overall accuracy of 56.1%, still suggesting that this practice of assessing sterility is inadequate [16]. Another source of surgical site infection is anesthesia ready time with a significantly higher infection rate in patients with anesthesia ready time longer than 1 hour [17].

However, there have been numerous studies on how to improve intraoperative sterility. Blom et al. showed bacterial penetration in all re-usable woven drapes while disposable drapes were impermeable [18]. Multiple studies recommend the use of disposable paper gowns due to a higher permeability of bacteria and thus a higher contamination rate in reusable gowns [19, 20]. Despite these recommendations, in 2019 Klaber et al. still found a bacterial contamination rate of 12% in 140 surgical gowns in different orthopaedic surgeries, ranging from 4.1% in hip surgery to 21.7% in spine and knee surgery [21].

With operation time the contamination rate of surgical gloves increases significantly, which is why change of surgical gloves before handling implants in order to minimize contamination is recommended [20]. In 43 knitted cotton outer gloves, Wichmann et al. found contamination above the estimated thresholds for implant-associated infection in a relevant proportion of examined gloves [22]. A study by Amirfeyz et al. focused on the contamination of theatre shoes and found significantly higher contamination of outdoor shoes compared to theatre shoes but no difference in samples taken from theatre shoes in the morning versus in the evening. The authors suggest that efficient floor cleaning therefore is important in order to keep shoe contamination levels low [23].

Another source of contamination during surgery is exposure of the used implants and instruments to air. Bible et al. obtained samples from 105 surgical trays at the end of an operation and compared their contamination to that of the paper outer wraps of the trays as well as to samples taken immediately after opening the tray. They were able to show a contamination rate of 2.0% of covered implants versus 16.7% of uncovered implants [24]. Dalstrom et al. did a comparable study in which they examined the contamination of 45 surgical trays at different time points after opening. Here, they were able to show that culture positivity directly correlated with the time the trays were openly exposed. Covering the surgical trays led to a significant reduction in contamination rates [25–27]. Using a guard in order to shield screws and other implants intraoperatively led to a reduction in bioburden compared to unguarded screws as shown by Agarwal et al. in 10 sterile packaged pedicle screws in 2019 [28]. Simply individually packaging screws might in fact lead to infection as suggested by Smith et al. who showed that in seven out of 50 cases, opening these screw-packets over a draped instrument table yielded bacterial growth. This however was not statistically significant [29].

Multiple studies have shown that optimizing the surgical environment leads to a reduced risk of surgical site infections. Positive correlations was found between the total colony-forming units (CFU) per operation and total traffic flow per operation, the number of persons present in the operating room, and the number of door openings [30, 31]. Additionally, in 207 surgeries Knobben et al. also showed that a number of disciplinary measures and the installation of a new laminar flow system lead to a decrease of instrument contamination from over 32.9 to 8.6% and a decrease in superficial surgical site infections from 11.4 to 1.4% [32]. The importance of air flow was also emphasized by other studies, which showed that laminar air flow or mobile laminar airflow units are able to reduce bacterial-carrying particles downstream of the surgical team and the mean bacterial count in the surgical wound [33–36].

Using ATP bioluminecence technology, Richard and Bowen found bioburden in 13 different operating room surfaces, including the OR preparation table and Bovie machine buttons. However, they did not correlate these findings with microbiology cultures or clinical infections [37]. Numerous studies have found that surgical tools such as supports used for patient positioning, pressure core prevention pads, and tourniquets were contaminated mostly with coagulase-negative staphylococci [38–40].

## Discussion

The literature evaluated in this systematic review shows that implant and instrument contamination before or during surgery is a well- and long-known problem, which may cause SSI and thereby lead to higher patient morbidity and longer patient hospitalization. While preoperatively mainly inadequate reprocessing was identified as a cause of implant contamination, intraoperatively, a variety of factors may threaten surgical asepsis. Most importantly, aseptic handling of all implants and instruments needs to be ensured. The abovementioned studies indicate that there is a need for improvement in the intraoperative handling of sterile medical products. Thus, different approaches need to be taken in order to prevent SSI.

As shown by multiple studies, an important factor of implant and instrument contamination is their exposure to air, which is why the duration of this exposure needs to be as short as possible and laminar airflow ventilation systems should be installed [24–27]. Additionally, surgical equipment including gloves and gowns play an important role in bacterial contamination. Several authors recommend the use of disposable gowns over reusable ones as they allow lower bacterial penetration [18–20]. To avoid the introduction of cutaneous bacteria from the patient’s skin into the surgical field, gloves should be changed right before handling implants. In their review on this subject, Agarwal et al. additionally list different techniques such as dipping implants in vancomycin or betadine, direct ultraviolet light exposure or covering implants with drapes, all of which yet need to be further examined regarding their ability to reduce SSI [41]. It is also important to point out that to date there is no convincing evidence regarding the relationship between contaminated implants or instruments and the actual development of SSI. Thus, despite its possibly detrimental effect on patient outcome, the extent of the problem remains unclear.

The included preoperative causes of implant contamination raise the question whether the commonly used process of sterilization is an effective way to decontaminate previously used medical products and to what extent screws, plates and other implantable items can be reprocessed at all. The abovementioned study by Agarwal et al. introduces two problems in the reprocessing procedure: 1) In the clinic, recommended reprocessing guidelines may be difficult to implement, especially considering the hospitals’ budgets. 2) Clinically feasible reprocessing may be insufficient in decontaminating medical products [7].

Due to these concerns regarding proper reprocessing of medical products, in the past few years prepackaged, sterile, single-use implants have become increasingly interesting. Not only does the repeated reprocessing of medical products lead to product contamination but the manufacturers’ guidelines often are impractical in the clinic. Thus, the reprocessing of certain medical implants has been banned in some countries such as Scotland and Japan and has been replaced by the use of disposable instruments [42].

However, many of the studies performed in this field are directly sponsored by the industry [43] or conducted by authors with conflicting interests [7]. Only a few small unbiased studies regarding the use of single-use instruments exist. In one of them, Litrico et al. showed in a prospective bi-centric study that the use of single-use surgical instruments in short instrumented spinal fusion surgery led to a reduction of SSI from 6% with reusable instrumentation to 2% with single-use instrumentation. At the same time, clinical outcomes were similar. The authors therefore concluded that wrapping screws and rods in sterile packs until insertion into the patient reduces infection rates by a reduction of exposure and of repetitive hospital sterilization [44].

As there still is a lack of larger studies and meta-analyses, so far no convincing evidence exists regarding the benefit of single-use instruments in orthopaedic surgery. Reprocessing of medical devices is, however, associated with risks and problems, which surgeons need to be aware of in order to guarantee patient safety. Here, more detailed and realizable instructions need to be provided by every manufacturer and training of reprocessing personnel needs to be improved. Most importantly however, ensuring a safe condition of the products used in surgery is not simply the manufacturers’ or the sterilization processing departments’ duty but also one of the personnel in the operating room.

While implant and instrument contamination plays a crucial role in the development of SSI, other risk factors need to be kept in mind to best prevent SSI. In spine surgery, these include not only patient-related factors such as age, comorbidities, smoking, or obesity but also surgery-related factors such as surgical approach, operation time, and blood loss [45–47]. To reduce SSI, the avoidance of implant and instrument contamination therefore needs to be accompanied by a multitude of strategies. As recommended by Anderson et al., patient selection and optimization prior to hospitalization including glycemic control and smoking cessation, perioperative antibiotics, skin antisepsis, and postoperative optimization of patient and wound care need to be implemented [48].

## Conclusion

The studies evaluated in this review provide evidence for multiple pre- and intraoperative causes for implant and instrument contamination. However, there have only been few studies on the relationship between contaminated implants or instruments and the actual development of SSI. Thus, the extent of the problem needs to be further investigated in order to provide specific solutions. Knowing of the frequency of contaminations and thereby the possibility of infections, more attention should be drawn to the condition of implants and instruments and known strategies should be implemented in daily clinical practice.

At the same time, the extension of single-use implants and instruments needs to be further studied. As shown by this review, errors in reprocessing are not the only causes of implant contamination and therefore it still needs to be shown that single-use implants actually lead to fewer postoperative infections. Thus, critical analyses not sponsored by the industry regarding the reduction of surgical site infection and regarding cost development are still needed before an unbiased recommendation for the use of these products can be given.

## Data Availability

Not applicable.

## Abbreviations

ART: Anesthesia ready time
ATP: Adenoside triphosphate
CFU: Colony-forming units
DG: Depth gauge
DV: Displacement ventilation
FMR: Flexible medullary reamer
LAF: Laminar airflow ventilation system
MLAF: Mobile laminar airflow ventilation system
OR: Operating room
SEM: Scanning electron microscopy
SSI: Surgical site infection
